# Laboratory selection of *Aedes aegypti* field populations with the organophosphate malathion: Negative impacts on resistance to deltamethrin and to the organophosphate temephos

**DOI:** 10.1371/journal.pntd.0006734

**Published:** 2018-08-20

**Authors:** Priscila Fernandes Viana-Medeiros, Diogo Fernandes Bellinato, Denise Valle

**Affiliations:** 1 Laboratório de Biologia Molecular de Flavivírus, Instituto Oswaldo Cruz (Fiocruz), Rio de Janeiro (RJ), Brasil; 2 Instituto Nacional de Ciência e Tecnologia em Entomologia Molecular (INCT-EM), Rio de Janeiro (RJ), Brasil; North Carolina State University, UNITED STATES

## Abstract

**Background:**

Resistance to pyrethroids and to the organophosphate temephos is widespread in Brazilian populations of the dengue vector, *Aedes aegypti*. Thereof, since 2009 Insect Growth Regulators are employed as larvicides, and malathion is used against adults.

**Methodology/Principal findings:**

We performed laboratory selection with malathion of two *A*. *aegypti* field populations initially susceptible to this organophosphate but resistant to temephos and deltamethrin. A fixed malathion dose inducing at least 80% mortality in the first generation, was used throughout the selection process, interrupted after five generations, when the threshold of 20% mortality was reached. For each population, three experimental and two control groups, not exposed to insecticides, were kept independently. For both populations, quantitative bioassays revealed, in the selected groups, acquisition of resistance to malathion and negative impact of malathion selection on deltamethrin and temephos resistance levels. In the control groups resistance to all evaluated insecticides decreased except, unexpectedly, to deltamethrin. Analysis of the main resistance mechanisms employed routine methodologies: biochemical and molecular assays for, respectively, metabolic resistance and quantification of the Na_V_ pyrethroid target main *kdr* mutations at positions 1016 and 1534. No diagnostic alteration could be specifically correlated with malathion selection, neither with the unusual deltamethrin increase in resistance levels observed in the control groups.

**Conclusions/Significance:**

Our results confirm the multifactorial character of insecticide resistance and point to the need of high throughput methodologies and to the study of additional field vector populations in order to unravel resistance mechanisms.

## Author summary

Dengue, Zika and chikungunya viruses affect millions of people worldwide. Due to the lack of specific antivirals or to the limited supply of vaccines, focus remains on the control of the main vector, *Aedes aegypti*. Although the importance of social participation in the elimination of *A*. *aegypti* breeding sites is increasingly recognized, chemical control is still an important component of vector control. The exaggerated use of insecticides results in the spread of resistance and, consequently, in the loss of their effectiveness. In Brazil, malathion is the last adulticide available to the control of *A*. *aegypti*, due to the widespread resistance to pyrethroids. In order to anticipate what could occur in the field, we exposed two vector populations to selection with malathion. Both malathion and temephos, a larvicide largely employed, are organophosphates; however, they are structurally distinct molecules and seem to elicit different resistance mechanisms. We confirmed this issue: selection with malathion had a negative impact on temephos resistance compared to groups reared without any insecticide. Indeed, the variety of responses of both vector populations to the various insecticides points to the participation of multiple resistance mechanisms and confirms previous assumptions regarding the difficulty of identifying diagnostic insecticide resistance mechanisms.

## Introduction

*Aedes aegypti* (Linnaeus, 1762) is the main vector of dengue, Zika and chikungunya viruses, which are currently serious public health problems in Brazil [1]. Specific medications are not available for any of these viruses. One dengue vaccine was recently approved but it confers only partial protection [2]; other vaccines against dengue and Zika viruses are yet in the clinical trials phase [3–5], and efforts in this regard for the chikungunya virus are just beginning [6]. Moreover, since 2016, Brazil faces a public health emergency with respect of yellow fever [7]. Although its transmission is silvatic, by *Haemagogus* and *Sabethes* mosquitoes [8], with the low vaccination coverage of the population there is an imminent risk of reurbanization of the virus, and *A*. *aegypti* is its main urban vector [9,10].

Except for the yellow fever virus, in the current scenario prophylactic measures depend on control initiatives aimed at decreasing vector density [11,12]. Although the most recommended and effective measures are the mechanical control and society awareness, chemical insecticides are still widely used tools. However, the intense exposure of vector and pest populations to insecticides can select characteristics and mechanisms that confer resistance to these xenobiotics [13]. Vector populations resistant to insecticides are a threat to the success of control programs that prioritize chemical control [14,15]. Thus, monitoring the susceptibility status of natural populations to the main employed insecticides is one of the pillars of vector control programs. This is a way of guiding control actions and preventing resistance of reaching a threshold considered of risk [12,16].

The organophosphate (OP) temephos has been used in the control of *A*. *aegypti* larvae in Brazil since 1967. Resistance to temephos, monitored in vector populations throughout the country, started in 2000–2001 and increases since then. In 2009, due to the spread of resistance [17], temephos ceased to be the first-choice larvicide, being replaced by chitin synthesis inhibitors (CSI) [15,18].

Similar to the control of larvae, OP were also used against adult mosquitoes up to 2001, when pyrethroids (PY) replaced them [19]. However, shortly thereafter, reduction of PY susceptibility of various field populations started to be identified [20,21], a process that resulted in the decision of interrupting the use of this class of insecticides in the control of *A*. *aegypti* adults in 2009 [18,22].

The Brazilian Ministry of Health (MoH) adopts only insecticides recommended by WHO [23] for the control of *A*. *aegypti*. Taking into account resistance of adults to PY insecticides, the only viable alternative for ultra low volume applications was malathion [23]. Although malathion is also an OP, such as temephos, the structure of both compounds is distinct: while temephos is a closed-chain phosphorothionate, malathion, an open-chain compound, is a phosphorodithioate, and bears two sulfur atoms attached to the central phosphorus, rather than just one [24]. Regarding the perspective of vector control, such differences are important, since they contribute to elicit different resistance mechanisms, as is the case with *Anopheles* mosquitoes, for example [25,26]. Indeed, in Brazil, in contrast to the widespread *A*. *aegypti* resistance to temephos, registers of altered malathion susceptibility were scarce, and only derived from qualitative bioassays [27,28]. When PY were replaced by malathion in the control of adults, quantitative assays confirmed the susceptibility of Brazilian natural *A*. *aegypti* populations to this OP (Braga TA, personal communication), corroborating the pertinence of its use in the field.

Taking this scenario into account, the anticipation of a potential malathion resistance event, as well as the elucidation of the possible mechanisms involved, would be of great value for the definition of rational strategies that could prolong this OP as a viable alternative for the control of *A*. *aegypti* adult specimens in Brazil. This study describes the selection with malathion, in the laboratory, of two *A*. *aegypti* field populations resistant to temephos and to the PY deltamethrin. Alterations in the malathion susceptibility profile were investigated, as well as the consequences of malathion selection on the susceptibility profile of the resulting samples to the main insecticides used in the country against the vector: temephos, the CSI diflubenzuron and deltamethrin. Collection of *A*. *aegypti* population from the municipality of Aracaju, at Sergipe State (SE) was made in 2012 when the MoH applied both temephos and CSI against larvae, and only PY to control adults. Crato mosquitoes, from Ceará State (CE), the higher resistant population, was collected in 2013 when temephos applications had just been replaced by CSI, and control of adults employed both PY and malathion. More details regarding the history of insecticides use in both localities are shown elsewhere [29]. Our major aim was to investigate if selection with malathion for a limited number of generations would be enough to alter the susceptibility profile of the exposed populations. In addition, it would be of interest to evaluate to what extent different vector populations, bearing distinct backgrounds regarding challenges with insecticides and other xenobiotics, would be impacted by malathion selection.

## Methods

### Mosquito lines

Sampling of *A*. *aegypti eggs* representative of the evaluated municipalities was done with ovitraps [27,30]. In the laboratory, mosquito colonies were started with 349 positive ovitraps from Aracaju / SE installed in 2012, and 272 positive ones from Crato / CE, installed in 2013 [29]. Egg hatching and larval rearing were carried out as routinely [27]. The resulting *A*. *aegypti* adults were then identified and used to obtain F1 eggs. Although the initial objective was to employ F1 specimens to characterize the resistance and the resistance mechanisms of the original populations [29] and also to proceed with the selection, the amount of eggs obtained was not enough. Therefore, F2 generation specimens were used in the selection procedure.

### Insecticides

In addition to malathion selection, the susceptibility status to the main insecticides applied by the Brazilian Dengue Control Program was analyzed. The study adopted technical grade compounds: the OP temephos (Pestanal; Sigma-Aldrich Brasil Ltda, São Paulo, SP, Brazil) and malathion (Cheminova Brasil Ltda, São Paulo, SP, Brazil), the PY deltamethrin (Pestanal; Sigma-Aldrich Brasil Ltda) and the CSI diflubenzuron (Pestanal; Sigma-Aldrich Brasil Ltda).

### Malathion selection

A malathion dose capable of killing about 80% of specimens in the first generation was used. This same dose was kept fixed until the mortality was only 20%. Three biological replicates of the selection (S1, S2 and S3) were done per population, without exchange of specimens between the replicates. For each population two control groups (C1 and C2) were also included, kept simultaneously in the same conditions, but without any contact with insecticides.

Malathion concentrations were defined in previous trials: 0.06229 mg/L for Aracaju and 0.09876 mg/L for Crato. For the first generation of selection, at least 5,000 larvae of each population were used per replicate, and 3,000 specimens were used to initiate subsequent generations of each independent experimental group. Synchronized larvae were used in the whole selection process: hatching of *A*. *aegypti* eggs was induced for at most one hour in a BOD incubator at 28°C. Larvae were then kept at 26 ± 2°C, at the density of 500 larvae per basin, in 1 L dechlorinated water and 1 g of grounded cat food (Friskies, Purina/Camaquã/RS) was provided. Under these conditions, larvae collected 72 hours after hatching were essentially L3 ones, and approximately 5% of the specimens had moulted to L4.

Basically, for each strain and each replica, several groups of 100 larvae, 72 hours old, were placed in plastic cylindrical basins (10 X 12 cm) with 250 ml of dechlorinated water and malathion ethanolic solution (for Aracaju and Crato samples, respectively 346 and 548.6 μl of a 45 mg/l malathion solution were employed) or equivalent volume of ethanol, in the case of control replicas. Larvae were fed with 0.2 g of grounded cat food added each three days. Exposure to malathion was continuous up to pupation. Pupae were daily transferred to cylindrical cardboard cages (17 X 18 cm). Upon eclosion, adults were fed *ad libitum* (except before the blood meal) with a 10% sugar solution, replaced three times a week and kept in a temperature and humidity-controlled insectary (26 ± 1°C; 80 ± 10% rh).

To obtain eggs, females were weekly deprived of the sugar solution for 18–24 hours, and anaesthetized guinea pigs were then offered as blood source according to the “Formulary for laboratory animals” [31].

After five generations, all replicates exposed to malathion exhibited less than 20% mortality in the presence of the insecticide, and the experiment was interrupted.

### Bioassays

Quantitative bioassays of larvae with temephos followed the parameters and procedures described by WHO [32]. Bioassays with diflubenzuron were made according to Martins et al. [33]. Adult bioassays with PY and malathion were done according to an adaptation of the insecticide impregnated papers methodology [34,35]. In all cases, three to four assays were performed for each population on different days. As an internal control, simultaneous trials were also carried out with the Rockefeller strain, a reference of insecticide susceptibility [36]. In each bioassay *A*. *aegypti* specimens were exposed to an insecticide spectrum of at least six concentrations. Larvae were exposed to temephos for 24 hours and to diflubenzuron until death or emergence of the last adult specimen of the control group, as previously defined by Braga et al. [37].

Adults were exposed to either deltamethrin or malathion for 1 hour and then transferred to a recovery chamber, with no insecticide, where they remained for another 24 hours, when mortality was recorded. For each assay with larvae, 320 to 640 specimens were used, totaling 960–1,920 specimens per insecticide tested by biological replicate (S1-S3, C1, C2). In the case of adults, 360–480 specimens were used per assay, corresponding to 1,080–1,440 specimens for each insecticide per biological replicate.

### Genotyping assays

The TaqMan method was used to identify *kdr* mutations in the Na_V_ gene in the post-selection samples, as previously described for pre-selection populations [29]. For each replica (C1, C2, S1, S2, S3), 30 individual males were used. Analysis of Aracaju samples was done with F7 specimens (offspring of the last generation of selection in the replicates S1-S3). In the case of Crato, analysis of C1 and C2 samples was done with F6, and S1-S3 replicates were investigated with F7 mosquitoes. Independent reactions were done for the substitutions Val1016Ile and Phe1534Cys. For each position, 1 μl, equivalent to 0.5% of the DNA content extracted from each specimen, was used in a 10 μl final reaction volume.

### Biochemical assays

One-day-old adult females were evaluated according to methodology adapted from WHO and the US Centers for Disease Control and Prevention (CDC) [38–40]. The same protocol, with some additional adaptations, was applied to larvae [41]. Activity of mixed function oxidases (MFO), esterases (EST) and glutathione S-transferases (GST) were quantified. Three substrates were used for ESTs: α-naphthyl, β- naphthyl and ρ-nitrophenyl acetates, related respectively to α-EST, β-EST and ρNPA-EST activities. For Acetylcholinesterase (Ace), the OP target site, both total activity (AChE) and the remaining activity after inhibition by propoxur (AChI) were evaluated. In order to calculate the specific enzymatic activities, total protein content of each sample was quantified using the Bio-Rad protein reagent (catalog number: 500–0006).

At least three assays were accomplished on different days for all enzymes. In each assay 40 individual specimens of each experimental replica were evaluated. Simultaneously, as an internal control of the assays, five specimens of the Rockefeller strain were also analyzed.

### Data analysis

In the case of bioassays, adult emergence inhibition (EI) or lethal concentrations (LC) were calculated, respectively for diflubenzuron and neurotoxic insecticides, by probit analysis using the Polo-PC software (LeOra Software, Berkeley, CA) [42]. Results of the quantitative assays were expressed as the ratio of resistance between the LC (or EI) of the experimental group under test and the Rockefeller equivalent measure (RR). Another index, 'selection ratio' (SR), was also calculated, by comparison of LC (or EI) values of C and S groups with their corresponding parental (P) population ones [29]. Additionally, we assessed the 95% confidence intervals overlap range between the post- and pre-selection LC of each experimental sample.

The criterion used in Brazil to classify the resistance status of *A*. *aegypti* populations to temephos [43] was also applied to the other insecticides evaluated in this study. According to this criterion, populations with RR_95_ higher than 3.0 are classified as resistant, and there is a recommendation for replacement of the insecticide compound used in the field.

Individual Na_V_ genotypes, as well as the allelic and genotypic frequencies of each population, were calculated based on the variations in positions 1016 and 1534, both on the same gene, as described elsewhere [44]. The 95% confidence intervals overlap range between the post- and pre-selection kdr allelic frequencies was also evaluated.

Enzymatic activities were classified according to a previously established criterion, based on the use of the 99th percentile of a reference strain as the cutoff point. In this case, references corresponded to the parental strains, from Aracaju and Crato [29]. After calculation of their 99th percentiles for each enzyme class activity, rates of corresponding specimens in the C and S groups above this value were estimated. The activities were classified as unaltered, altered or highly altered in cases where these rates were respectively less than 15, between 15 and 50, or above 50% [43,38]. In particular, in the case of the Ace inhibition assay with propoxur (AChI), the WHO criterion establishes that remaining activity exceeding 30% is indicative of resistance to OP insecticides [40]. All enzyme activity profiles were also compared using the Kruskal Wallis nonparametric test and the Dunn's multiple comparison post test (using the graphpad prism version 5.0).

### Ethics statement

The blood feeding was done according to the Brazilian guidelines described in “The Brazilian legal framework on the scientific use of animals” [45], supported by a protocol approved by the "Ethics Committee in the Study of Animals" (CEUA/Fiocruz 2008), licenses L-011/09 and LW-20/14.

## Results

### Selection

**Fig 1:**
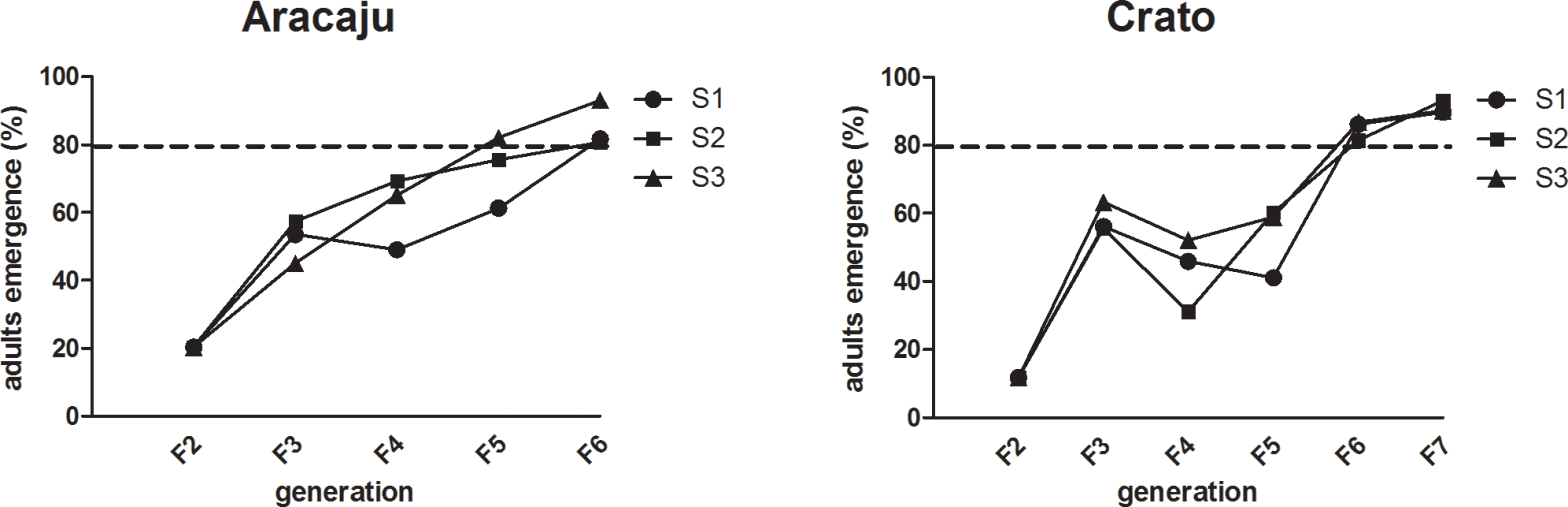
Survival rate of two *Aedes aegypti* populations subjected to selection with a fixed dose of malathion. Exposure to the insecticide was continuous, between L3 and pupal phases. The x-axis indicates the generations of laboratory rearing.

In the first generation of selection with a fixed malathion dose, respectively 20.4 and 11.8% of Aracaju and Crato individuals survived. In the course of the selection process, the Aracaju survival percentage increased more evenly than that of Crato. Nevertheless, both populations reached the threshold of 80% survival (dotted line in Fig 1), defined as the time of selection interruption, in the 5^th^ generation (F6). For the population of Crato, exposure to malathion was repeated with one additional generation, when 90.9% of survival was detected. After that, the potential impact of laboratory selection on the resistance status of malathion itself (in larvae and adults) and on other compounds used in the *A*. *aegypti* control in the country was assessed. It is ought to mention that mortality in the control groups did not exceed 1% during the whole process.

### Insecticide resistance: Bioassays

Table 1 summarizes the impacts of malathion selection and of adaptation to laboratory breeding on the RR profile to several insecticides employed in *A*. *aegypti* control. In order to facilitate a direct comparison, results of the original evaluations with the pre-selected populations [29] were included. Bold values in Table 1 point to resistance when the Brazilian MoH classification criteria, previously defined for temephos, is used (RR_95_ > 3.0, see Methods). Detailed results are presented in S1–S5 Tables, including comparison of mortality values obtained for C and S groups with their corresponding parental (P) populations (the 'selection ratio', or SR analysis).

**Table 1:** Resistance status of *A*. *aegypti* larvae and adults from Aracaju/SE and Crato/CE to the insecticides used by the Brazilian MoH. Samples before and after the malathion selection process are shown.

			malathion (larvae)		temephos		diflubenzuron		malathion (adults)		deltamethrin	
population	sample	generation	RR_50_	RR_95_	RR_50_	RR_95_	EI_50_	EI_95_	RR_50_	RR_95_	RR_50_	RR_95_
Aracaju	P	F1	2.6	2.9	11.2	**12.9**	1.6	1.7	1.6	1.8	14.3	**17.8**
	C1	F7	2.4	2.6	8.7	**11.5**	1.4	1.3	1.7	2.4	40.7	**60.2**
	C2		2.3	2.5	8.3	**12.9**	1.2	1.6	1.6	2.6	41.8	**90.5**
	S1	F7	2.4	**3.1**	5.8	**9.6**	1.4	1.8	1.8	2.4	11.6	**23.0**
	S2		3.5	**4.0**	7.5	**10.3**	1.4	1.6	2.0	2.7	9.7	**17.2**
	S3		2.9	**3.8**	8.5	**11.8**	1.6	1.9	1.9	2.4	10.9	**16.4**
Crato	P	F2	2.4	2.8	23.2	**64.8**	1.6	1.8	1.6	2.0	37.0	**51.6**
	C1	F6	2.3	2.6	19.2	**43.9**	1.3	1.3	1.8	2.7	39.6	**91.2**
	C2		2.2	2.5	13.5	**40.4**	1.2	1.2	1.9	2.5	40.8	**75.3**
	S1	F7	5.3	**5.9**	8.1	**26.6**	1.5	1.8	2.5	**3.2**	28.3	**35.0**
	S2		4.7	**5.1**	9.1	**45.7**	1.8	1.7	2.5	**3.7**	26.4	**41.1**
	S3		4.8	**5.3**	12.4	**44.3**	1.6	1.7	2.3	**3.4**	32.2	**37.7**

P: results of pre-selection populations, previously described by Viana-Medeiros et al. [29]; (C1, C2): samples kept in the laboratory, without insecticides; (S1-S3) samples after selection of larvae with malathion. In all cases, the classification criterion employed for temephos in Brazil was applied [43,15]: samples with RR_95_ greater than 3.0 (shown in bold) are considered resistant. The Rockefeller strain was used as an insecticide susceptibility control. For effective doses and confidence intervals see S1–S5 Tables.

Both analytical approaches pointed out the effectiveness of the laboratory selection promoted in the study. Although SR data are slighter than RR ones: (a) both malathion RR and SR increased in the larvae of virtually all selected groups—malathion SR values increased in the range of 50% and more than twice for, respectively, Aracaju and Crato selected larvae; (b) in general, there was no overlap of CL values between C and S groups, pointing to a sound effect of malathion selection (S1 Fig); (c) for Crato, the selective pressure reverberated in the adult stage to the point that it could also be classified as resistant after selection, when the 'MoH RR_95_ criterion' was used. Malathion SR values increased in the range of 50% for Crato adults. Groups maintained without insecticide showed a slight reduction of malathion RR in the larval stage (which were already low), and a discrete increase in the adult stage, while SR revealed a slight increase in both stages (S1 and S2 Tables). In general, for each population, C replicas, as well as S ones, had an equivalent performance, although reared independently.

Laboratory rearing in the absence of insecticides (groups C1, C2) was enough to slightly reduce the initial temephos resistance levels exhibited by parental populations. Selection with malathion did not induce cross-resistance with temephos, although both are OP (Tables 1 and S3). In contrast, temephos resistance decrease was more noticeable in malathion selected groups than in the control ones–both RR and SR indexes suggested such a trend. In particular, temephos resistance reduction was more pronounced in Crato, the population with the highest initial RR.

When the present study started, diflubenzuron had been recently introduced in the country to control *A*. *aegypti* larvae. Resistance indexes for this CSI compares adult emergency inhibition rates (EI), and not lethal concentrations. When compared to the original populations, although some differences in RR and SR for DFB were observed between C and S groups, all values obtained were considered compatible with a susceptible status (Tables 1 and S4).

All samples were considered highly resistant to deltamethrin. Surprisingly, the simple laboratory rearing, in the absence of any insecticide (groups C1, C2), exacerbated PY resistance levels in both populations (Tables 1 and S5). In particular, the SR index confirmed the marked deltamethrin resistance increase in the control groups, mainly in Aracaju (S5 Table). This increase in deltamethrin resistance, however, was not observed in the S1-3 groups, selected with malathion, independently of the analytical method adopted (RR, SR and 95% confidence limits overlapping). In opposition, malathion selected groups, and particularly Crato mosquitoes, exhibited reduction of deltamethrin resistance levels.

In general, when compared to the parental samples and as judged by slope values, homogeneity of all evaluated groups remained unaltered or increased (S1–S5 Tables). Only with deltamethrin bioassays there was a trend towards increased heterogeneity (except for Crato S groups). In this regard, the unusual and specific increase in deltamethrin resistance, noted in the control groups, should be considered.

### Insecticide resistance mechanisms: *kdr* mutations genotyping

Table 2 shows the *kdr* genotypes at positions 1016 and 1534 of the Na_V_ gene, the target site of PY insecticides. The 'wild type' susceptible allele, '1016 Val^+^ + 1534 Phe^+^', was called 'S'. The allele mutated only at position 1534 (1016 Val^+^ + 1534 Cys^*kdr*^) was named 'R1' and the other, with mutations at both positions (1016 Ile^*kdr*^ + 1534 Cys^*kdr*^), 'R2'. Mutation exclusively at position 1016 was not found. The three alleles were detected in Aracaju and Crato. Since *kdr* mutations at positions 1016 and 1534 are recessive, PY resistance mediated by such changes is only expressed in homozygous individuals: R1R1, R1R2 and R2R2 [44] (highlighted in bold and summed in column ‘resistant genotypes’ in Table 2).

**Table 2:** Changes in the voltage-gated sodium channel (AaNa_V_), target of pyrethroids, in Aracaju and Crato, during the malathion selection process.

population	sample	G*	genotypic frequencies							resistant genotypes**	allelic frequencies		
			*n total*	SS	SR1	R1R1	SR2	R1R2	R2R2		S	R1	R2
Aracaju	P	F0	*30*	0.20	0.03	**0.03**	0.33	**0.10**	**0.30**	***0*.*43***	0.38 (0.26–0.51)	0.10 (0.02–0.18)	0.52 (0.39–0.65)
	C1	F7	*30*	0.10	0.13	**0.03**	0.37	**0.27**	**0.10**	***0*.*40***	0.35 (0.23–0.47)	0.23 (0.13–0.34)	0.42 (0.29–0.54)
	C2		*30*	0.23	0.10	**0.00**	0.47	**0.10**	**0.10**	***0*.*20***	0.52 (0.39–0.64)	0.10 (0.02–0.18)	0.38 (0.26–0.51)
	S1	F7	*29*	0.17	0.17	**0.00**	0.52	**0.07**	**0.07**	***0*.*14***	0.52 (0.39–0.65)	0.12 (0.04–0.20)	0.36 (0.24–0.49)
	S2		*30*	0.27	0.07	**0.07**	0.40	**0.13**	**0.07**	***0*.*27***	0.50 (0.37–0.63)	0.17 (0.07–0.26)	0.33 (0.21–0.45)
	S3		*30*	0.10	0.23	**0.03**	0.37	**0.20**	**0.07**	***0*.*30***	0.40 (0.28–0.52)	0.25 (0.14–0.36)	0.35 (0.23–0.47)
Crato	P	F2	*28*	0.21	0.32	**0.18**	0.14	**0.11**	**0.04**	***0*.*32***	0.45 (0.32–0.58)	0.39 (0.26–0.52)	0.16 (0.06–0.26)
	C1	F6	*28*	0.14	0.32	**0.14**	0.32	**0.07**	**0.00**	***0*.*21***	0.46 (0.33–0.59)	0.34 (0.22–0.46)	0.20 (0.09–0.30)
	C2		*30*	0.50	0.30	**0.10**	0.03	**0.07**	**0.00**	***0*.*17***	0.67 (0.55–0.79)	0.28 (0.17–0.40)	0.05 (0–0.11)
	S1	F7	*29*	0.21	0.31	**0.10**	0.35	**0.03**	**0.00**	***0*.*14***	0.53 (0.41–0.66)	0.28 (0.16–0.39)	0.19 (0.09–0.29)
	S2		*30*	0.13	0.53	**0.00**	0.20	**0.10**	**0.03**	***0*.*13***	0.50 (0.37–0.63)	0.32 (0.20–0.43)	0.18 (0.09–0.28)
	S3		*29*	0.38	0.21	**0.14**	0.07	**0.14**	**0.07**	***0*.*35***	0.52 (0.39–0.65)	0.31 (0.19–0.43)	0.17 (0.08–0.27)

* Generation

The CI95% is under parentheses.

The alleles S, R1 and R2 refer to Na_V_ positions 1016 and 1534: 1016 Val^+^ + 1534 Phe^+^ (S, susceptible), 1016 Val^+^ + 1534 Cys^*kdr*^ (R1) and 1016 Ile^*kdr*^ + 1534 Cys^*kdr*^ (R2). The homozygous genotypes of these recessive *kdr* mutations are highlighted in bold. For each group, 28 to 30 adult males were evaluated, as indicated in the '*n total*' column.

Column resistant genotypes**: sum of the frequencies of the genotypes R1R1 + R1R2 + R2R2, all of them homozygous for *kdr* mutations. The 95% confidence intervals of allelic frequencies are in parentheses. Frequencies of the mutations at positions 1016 and 1534, alone, are shown in S6 Table.

P: previously presented Aracaju [44] and Crato [29] pre-selection data.

In Crato the wild-type (S) allele remained as the most frequent, followed by R1, a pattern kept in all sample groups from that locality. On the other hand, R2 was the most frequent allele in Aracaju, followed by the S allele but this arrangement tended to reverse after laboratory rearing. Regarding the original populations, in both cases, an increase in the frequency of the S allele was observed in almost all samples, selected with malathion or simply reared in the laboratory, without insecticides (note the IC 95% overlap range in S2 Fig).

The initial *kdr* genotype frequencies, exhibiting PY resistance, were 43% for Aracaju and 32% for Crato ('*resistant genotypes*' column in Table 2). In almost all cases, in both groups C and S, such frequencies decreased during the selection or rearing processes.

### Insecticide resistance mechanisms: Metabolic resistance and OP target

Tables 3, 4 and S7 show the activity profiles of the major classes of detoxification enzymes in larvae and adults of Aracaju and Crato mosquito populations. Acetylcholinesterase activity, target site of OP, was also quantified; in this case the total activity (AChE) and its profile of inhibition by propoxur (AChI) are presented. Tables 3 and 4 depict, respectively, the percentage of Aracaju and Crato individuals with activity above the 99^th^ percentile of each corresponding Parental strain; S7 Table compares median values of the same samples, using the Kruskal-Wallis nonparametric test.

**Table 3:** Quantification of detoxifying enzymes and acetylcholinesterase activity in Aracaju *A*. *aegypti* exposed to selection with malathion—Comparison with the 99^th^ percentile of the parental population.

samples			AChI	AChE	MFO	α-Est	β-Est	*ρnpa-Est*	GST
**Aracaju (larvae)**			*Par 99*^*th*^ *p*^*1*^						
	Par	F1	19.66	0.10	92.85	33.14	48.80	6.98	1.27
			*% > Par 99*^*th*^ *p*^*2*^						
	C1	F7	**1**	**0**	**0**	**0**	**0**	**0**	**2**
	C2		**1**	**0**	**0**	**0**	**0**	**0**	**1**
	C1+C2		**1**	**0**	**0**	**0**	**0**	**0**	**2**
	S1	F7	**1**	**3**	**0**	**1**	**0**	**0**	**9**
	S2		**0**	**3**	**0**	**0**	**0**	**1**	**0**
	S3		**1**	**3**	**4**	**0**	**3**	**3**	**0**
	S1+S2+S3		**1**	**3**	**1**	**0**	**1**	**1**	**3**
**Aracaju (adults)**			*Par 99*^*th*^ *p*						
	Par	F1	27.93	0.21	70.71	14.86	14.78	12.07	1.61
			*% > Par 99*^*th*^ *p*						
	C1	F7	**0**	**1**	**75**	**0**	**0**	**0**	**30**
	C2		**0**	**0**	**80**	**0**	**0**	**0**	**23**
	C1+C2		**0**	**1**	**78**	**0**	**0**	**0**	**27**
	S1	F7	**0**	**2**	**45**	**0**	**0**	**0**	**5**
	S2		**0**	**0**	**33**	**0**	**0**	**0**	**7**
	S3		**0**	**9**	**71**	**0**	**0**	**0**	**10**
	S1+S2+S3		**0**	**3**	**50**	**0**	**0**	**0**	**8**

Enzyme activities were classified according to the percentage of individuals with activity above the 99^th^ percentile of the corresponding parental population [29]: <15%, between 15 and 50%, and> 50%, being considered normal (in light gray), altered (dark gray), and heavily altered (in black), respectively. All control groups were clustered (‘C1+C2’ line), as well were the selected groups (‘S1+S2+S3’ line).

(1): the cutoff adopted.

(2): the percentage of individuals with activity above the cutoff point.

**Table 4:** Quantification of detoxifying enzymes and acetylcholinesterase activity in Crato *A*. *aegypti* exposed to selection with malathion—Comparison with the 99^th^ percentile of the parental population.

samples			AChI	AChE	MFO	α-Est	β-Est	*ρnpa-Est*	GST
**Crato (larvae)**			*Par 99*^*th*^ *p*^*1*^						
	Par	F1	21.67	0.09	62.43	24.68	45.86	5.51	1.98
			*% > Par 99*^*th*^ *p*^*2*^						
	C1	F7	**3**	**1**	**9**	**15**	**1**	**1**	**0**
	C2		**3**	**0**	**0**	**9**	**0**	**1**	**0**
	C1+C2		**3**	**1**	**4**	**12**	**1**	**1**	**0**
	S1	F7	**0**	**11**	**8**	**15**	**5**	**3**	**0**
	S2		**0**	**4**	**4**	**27**	**0**	**1**	**0**
	S3		**0**	**5**	**10**	**26**	**13**	**3**	**0**
	S1+S2+S3		**0**	**7**	**7**	**23**	**6**	**2**	**0**
**Crato (adults)**			*Par 99*^*th*^ *p*						
	Par	F1	21.76	0.19	51.16	9.50	9.58	6.56	1.92
			*% > Par 99*^*th*^ *p*						
	C1	F7	**9**	**0**	**100**	**38**	**42**	**85**	**0**
	C2		**3**	**1**	**100**	**26**	**32**	**60**	**0**
	C1+C2		**6**	**1**	**100**	**32**	**37**	**73**	**0**
	S1	F7	**6**	**1**	**80**	**40**	**27**	**73**	**3**
	S2		**6**	**0**	**100**	**61**	**56**	**99**	**0**
	S3		**0**	**0**	**99**	**37**	**26**	**54**	**3**
	S1+S2+S3		**4**	**0**	**92**	**46**	**36**	**75**	**2**

Details as in Table 3 legend.

For both populations more changes and higher activities were noted in adult mosquitoes, compared to the larval stage, in all C and S groups. The exceptions were α-EST and β-EST activities, which are higher in larvae (S7 Table).

In general, no differences were observed between control groups and those exposed to malathion which could be classified as diagnostic of the selected resistance.

In relation to the parental samples, Aracaju adult females tended to present higher MFO and GST activities, the later being revealed by both criteria in control samples (Tables 3 and S7) but detected only after comparison of median values in S ones (S7 Table). In Crato, marked alterations of all classes of enzymes were also observed in both C and S females, when compared to the Parental population; however, GST alterations were more attenuated in relation to the other enzyme classes, being detected only by medians comparison (Tables 4 and S7).

In the larval stage, no metabolic alterations were detected in Aracaju C and S samples, compared to the original population. In Crato larvae, α-EST EST activity was consistently enhanced and MFO alterations were noted in some samples when median values were compared, particularly in the malathion selected ones.

Total activity of the OP target site, AChE, remained equivalent to the original samples. We also investigated Ace using the WHO susceptibility criterion: inhibition of 70% or more of Ace's activity with propoxur (AChI); in all cases the results are indicative of a susceptible enzyme (Tables 3, 4 and S7). According to this criterion, Ace of both parental Aracaju and Crato populations has a susceptible profile.

## Discussion

This study deals with the response to laboratory rearing and to selection with malathion of two *A*. *aegypti* field populations. Their resistance status to the main insecticides employed by the Brazilian MoH as well as the potentially associated resistance mechanisms were evaluated.

The outcome of malathion selection was considered consistent, since resistance to this OP increased in the three biological replicates of both Aracaju and Crato. This increase was seen in all the adopted analytical approaches—comparison with the Rockefeller susceptible strain or with the parental generation. Although comparison with the parental population (SR values) showed more slight increases than comparison with the susceptible reference strain (RR), both selection efficacy and the functional significance of this result are confirmed since there is no overlap of CL ranges between the selected groups and the control samples, nor with the parental ones. Considering also the initial and final mortalities of the malathion selection experiment, which ranged from 80 to less than 20% in the six independently selected groups (Fig 1), we can infer that malathion selection appears to have had a relevant biological impact.

The magnitude of such increases in resistance levels was also compatible with other records in the literature that subjected *A*. *aegypti* populations to different insecticides for a small number of generations [46,47].

Although selection has increased malathion resistance levels, resistance to temephos tended to decrease in both populations. Such reduction was more prominent in the samples selected with malathion than in the control groups. This situation was, to some extent, expected, considering the molecular structures of both insecticides and other reports in the literature [25,48–50]. Collectively, such data reinforce the idea that these two organophosphates elicit different mechanisms of resistance [25,50,51]. This also occurs, for example, with species of the genera *Anopheles* and *Culex*: resistance to temephos has been found to be derived from the copy number amplification of esterases genes, culminating in a greater number of available enzyme molecules [51,52]; on the other hand, malathion metabolic resistance is related to alterations in the coding region of a particular esterase, called 'malaoxonase', resulting in a more efficient enzyme [26,53].

In fact, the two OPs differ in chemical structure, as stated in the Introduction. Such differences appear to result in the extremely slow hydrolysis of temephos, via esterases, a process that may last for days. In this case, it is even considered that temephos is sequestered [48,50]. As a consequence, esterase-mediated resistance to OP compounds, in general, would occur due to an increase in the amount of enzyme molecules, as is the case with *Culex* mosquitoes [51,54]. In our study, alterations in some specific resistance mechanisms were observed. However, such changes were not able to elucidate all the complexity observed in the responses of the different sample groups of Aracaju and Crato, related to the evaluated insecticides.

In the samples here evaluated, changes in the activity of enzymes related to metabolic resistance were noted, particularly in adult specimens of the vector. However, no difference was observed that was exclusive to the replicates submitted to selection, in both populations. This result suggests the participation of different molecular species and points to the need of high-performance methodological approaches in the identification of resistance mechanisms. However, this type of evaluation is certainly beyond the scope of the tests used in routine resistance monitoring [12,43,55,56].

Regarding Ace, target of OP insecticides, no significant changes were noted neither in total activity (AChE) nor in inhibition of activity (AChI). In general, AChI results were concordant for the two classification criteria employed, WHO [40] and Valle et al. [38]. Therefore, the study of the OP target revealed no alterations likely to explain the decrease in malathion susceptibility in the samples exposed to this insecticide.

Malathion selection appears to have had a negative impact on deltamethrin resistance, similar to what happened with temephos status of malathion selected mosquitoes. In both cases, temephos and deltamethrin, the RR reduction was more prominent in the malathion selected groups than in the control ones. One potential reason is the selection of malathion resistance mechanisms, in detriment of others, previously present in the original samples and related to resistance to temephos and deltamethrin. In other words, in the absence of changes in Ace, the target of OP, this scenario may result from the deviation of metabolic resources, in the scope of resistance mediated by detoxifying enzymes [57,58]. It is worth mentioning that decrease of PY resistance levels in the malathion selected groups was followed by the maintenance (in Crato) ou slight reduction (in Aracaju) of the *kdr* resistant homozygotes frequency.

Unexpectedly, a marked increase in deltamethrin resistance levels was observed in the control groups, maintained without any insecticide, relative to the original samples, in both populations. This occurred despite a slight decrease in the *kdr* frequencies, a known mechanism of PY resistance. This decrease in *kdr* mutations rate is somewhat expected since there are indications that, in the absence of PY, *kdr* mutations do not offer an adaptive advantage; instead, this feature is related to a significant evolutionary cost [59]. Of course, the possibility of contamination cannot be completely ruled out, although the whole rearing process has been tracked, as well all bioassays steps. However, taken together, such data suggest that (a) the *kdr* mutation frequency assessment should not be used as a single marker of PY resistance, at least in *A*. *aegypti*, and (b) additional mechanisms capable of conferring PY resistance were selected during the laboratory procedures.

In general, selection experiments performed under laboratory conditions tend to privilege multiple low-effect mechanisms, as would be expected in the field, according to assumptions of natural selection [60]. However, in the field, application of insecticides seems to be just one amongst the many challenges faced by mosquitoes. *Aedes aegypti*, as a generalist insect, can also deal with the presence of pollutants or secondary plant metabolites in its larval breeding sites. This condition could bestow a pre-adaptive advantage regarding the insecticide challenge and justify the early detection of resistance to newly introduced compounds, for instance, in the context of vector control programs [20,21]. In fact, some metabolites and pollutants were able to induce a significant increase in the expression of MFO enzymes in *A*. *aegypti*, corroborating such pre-adaptability for insecticide resistance [61].

As expected, higher changes in resistance levels were observed in the larval stage, submitted to selection. However, the metabolic resistance components evaluated were more altered in adults. The methodology used here for the quantification of resistance mechanisms is the approach of choice in the routine of the resistance monitoring of vector populations. However, this methodology has known limitations: it uses general substrates, which reveal classes of enzymes and not specific molecular entities. This means that eventual changes in some particular enzyme of a given class can be masked by the pool of enzymes acting on that substrate. In addition, it is possible that other mechanisms, not investigated by this approach, are in operation.

Evaluation of metabolic activities in adult Aracaju females indicated a potential participation of GST enzymes in the increased deltamethrin resistance: after comparison with both criteria, parental 99th percentile and the nonparametric statistics, higher GST activity was detected in Aracaju samples kept without insecticide—precisely those with a marked increase of PY resistance levels. Some studies suggest relation between GST and PY resistance of *A*. *aegypti* populations from different geographic origins [43,62,63]; others have even characterized this association [49,64]. However, it is worth noting that in adult females from Crato control groups, although also bearing an increased deltamethrin resistance, the change in GST was not prominent, being only detected through medians comparison. It is well known that insecticide resistance has a multifactorial character. There are several recent examples and there is growing understanding that different populations of the same vector species can select different resistance mechanisms against the same class of insecticides. This can derive from several parameters, intrinsic and extrinsic. Among the former are the genetic background and previously selected mechanisms, while insecticide selection pressure intensity and sources are among the extrinsic factors [15,46,63,65,66].

Although malathion is an adulticide, we opted to employ larvae in the selection procedure for operational reasons. On one hand, we were interested in carrying out this analysis in a timely manner to respond to the management of resistance in the country; on the other hand however, the technical difficulty of calibrating selection trials with adults is greater than with larvae. Selection with larvae was then a cost-effective way in which we decided to invest in order to ensure that all specimens would be exposed to malathion in a way as controlled as possible. Culicidae males are smaller and develop faster than females. In this case, the ideal selection with adults would require different experiments, with distinct malathion concentrations for males and females. Furthermore, in bioassays with adults, mainly with the available impregnated surfaces, it is difficult to control the level of exposure, since contact with insecticides occurs only during landing. Several studies report the use of larvae in selection experiments with adulticides and, in general, this apparent inconsistency is not even mentioned in the texts (examples in [67–78]). Despite this, we are aware that the selection of larvae with malathion here shown may not represent the field situation reliably, and the results may have limited value in defining resistance management recommendations. For example, there is at least one report of the development of resistance to the adulticide deltamethrin that was more effective in larval selection, compared to adults [79]. In another study, selection of adult females with the same PY also resulted in only mild resistance status alteration of a Brazilian *A*. *aegypti* population [80].

It is possible that adaptation to laboratory rearing, in the absence of insecticides, has favored, or selected, one or more mechanisms relevant to the response to PY. Unlike changes in Na_V_ (*kdr*), such mechanisms could have borrowed advantage to specimens carrying them. This might have occurred even in the absence of selective pressure and under optimal rearing conditions. If this is the case, malathion selection may have hampered the expression of such mechanisms in some way: lower levels of PY resistance were detected in the selected specimens compared to the control groups.

In general, it is expected that laboratory rearing, without selective pressure (as was the case with the control groups herein), will increase homogeneity regarding insecticide susceptibility [81]. However, our data indicate that this relationship, between absence of selective pressure and reduction of heterogeneity, although frequent, cannot be generalized. Slope is often a parameter put aside in the insecticide resistance monitoring of disease vectors. Few studies mention the heterogeneity of the evaluated samples, or often fail to inform details (reports we detected that mention slope results: [27,28,37,56,82–88]). In the present study, homogeneity increase was noted in most groups, regardless the insecticide challenge, probably due to rearing under laboratory-controlled conditions. The main exception occurred with deltamethrin evaluation: control groups of both populations presented greater heterogeneity related to the PY. And it was precisely for deltamethrin that an unusual and marked resistance increase was detected in these control groups.

Laboratory selection of samples from two Brazilian *A*. *aegypti* natural populations with the OP malathion was attained. In both cases, alterations in the resistance profiles related to other insecticides employed in the country against *A*. *aegypti* were also observed. No cross-resistance was detected between malathion and temephos, also an OP, nor between malathion and deltamethrin, a PY. On one hand, malathion selection had a negative effect on resistance to other insecticides. On the other hand, maintenance of both populations in an insecticide-free environment resulted in an unexpected increase in resistance to deltamethrin. However, the methodological procedures routinely adopted by the control programs did not reveal the potential resistance mechanisms associated, in each case. This scenario suggests the pertinence of adopting high throughput investigation approaches which we are, in fact, providing with the material presented here.

Our data point to the feasibility of applying diflubenzuron and malathion in *A*. *aegypti* control in Brazil, respectively against larvae and adults. A larvicidal rotation scheme, as already recommended by the MoH, could certainly contribute to preserve diflubenzuron (or another CSI) in the field as a viable strategy. Unfortunately, a similar approach with malathion is not possible since all the alternative available adulticides are PY compounds [23], and *A*. *aegypti* resistance against this class of insecticides is widespread throughout the country [20,21,29,44,56]. Finally, considering the insecticides employed by control programs, our results reinforce the relevance of investigating the susceptibility status, and the associated mechanisms, of more field *A*. *aegypti* populations. This policy could subsidize the rational choice of the compounds and collaborate to keep the feasibility of the few available compounds.

## Supporting information

S1 Fig Malathion susceptibility levels (mg/l). For each evaluated population and development stage, the 95% confidence limits of both lethal concentrations, LC_50_ and LC_95_, were plotted in order to estimate overlapping ranges among samples reared in the laboratory (C, S for control and malathion selected, respectively) and the parental (P) ones.
(TIF)

S2 Fig *kdr* allelic frequencies. The 95% confidence limit of each one of the alleles S (1016 Val + 1534Phe), R1 (1016 Val + 1534Cys) and R2 (1016Ile + 1534Cys) was plotted for both Aracaju and Crato mosquitoes S: green, R1: yellow, R2: pink. In the x-axis, P, C_1-2_ and S_1-3_ refer to, respectively, Parental, Control and Selected samples.
(TIF)

S1 Table Details of malathion bioassays performed with *Aedes aegypti* larvae samples. Results generated by probit analyses. Results of bioassays with the Rockefeller strain tested simultaneously to the experimental and control groups are also shown. SR: ‘Selection Rate’, ratio between the LC (or EI) values of C and S groups and their corresponding parental population (P). LC: lethal concentration. The 95% confidence interval is shown.
(PDF)

S2 Table Details of malathion bioassays performed with *Aedes aegypti* adult samples. Legend as in S1 Table.
(PDF)

S3 Table Details of temephos bioassays performed with *Aedes aegypti* larvae samples. Legend as in S1 Table.
(PDF)

S4 Table Details of diflubenzuron bioassays performed with *Aedes aegypti* larvae samples. Legend as in S1 Table. EI: Emergence inhibition.
(PDF)

S5 Table Details of deltamethrin bioassays performed with *Aedes aegypti* adult samples. Legend as in S1 Table.
(PDF)

S6 Table Allelic and genotypic frequencies of *A*. *aegypti* Na_V_ gene at positions 1016 and 1534, shown separately. (PDF)

S7 Table Quantification of detoxifying enzymes and acetylcholinesterase activity in Aracaju and Crato *A*. *aegypti* exposed to selection with malathion—Comparison of median values. Samples' median values were compared with the corresponding medians of parental strains [29] using the Kruskal-Wallis test. (*): significantly different values (p<0.01). n^1^: number of individuals considered in the analysis. med^2^: median of enzymatic activities.
(PDF)
