# Supplementary figures and images for: Laboratory selection of Aedes aegypti field populations with the organophosphate malathion: Negative impacts on resistance to deltamethrin and to the organophosphate temephos
Source: PLoS Negl Trop Dis. 2018 Aug 20;12(8):e0006734. doi: 10.1371/journal.pntd.0006734 (PMC6128625; doi:10.1371/journal.pntd.0006734)

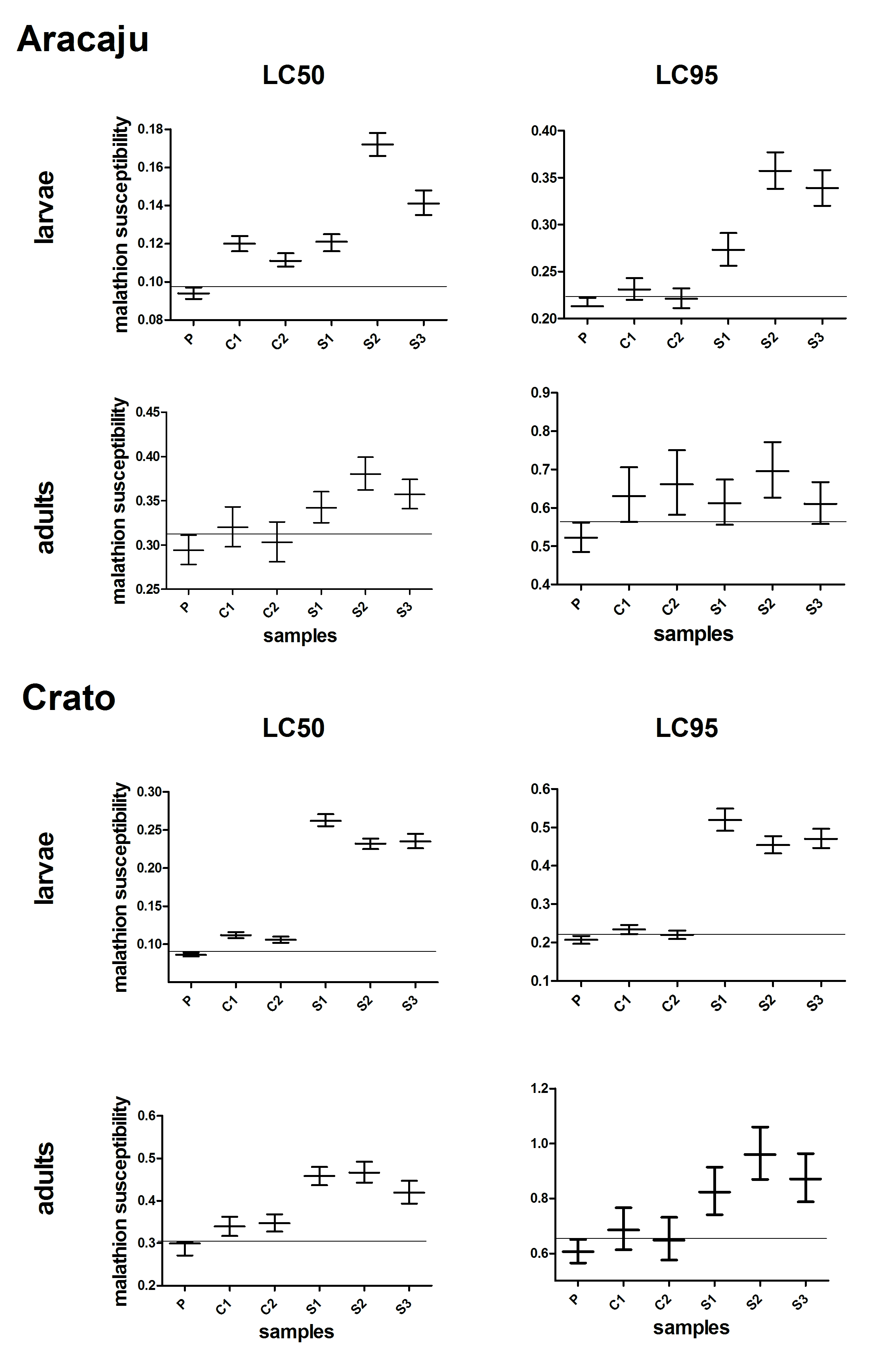

Supplement: S1 Fig — For each evaluated population and development stage, the 95% confidence limits of both lethal concentrations, LC50 and LC95, were plotted in order to estimate overlapping ranges among samples reared in the laboratory (C, S for control and malathion selected, respectively) and the parental (P) ones. (TIF) [file pntd.0006734.s001.tif]

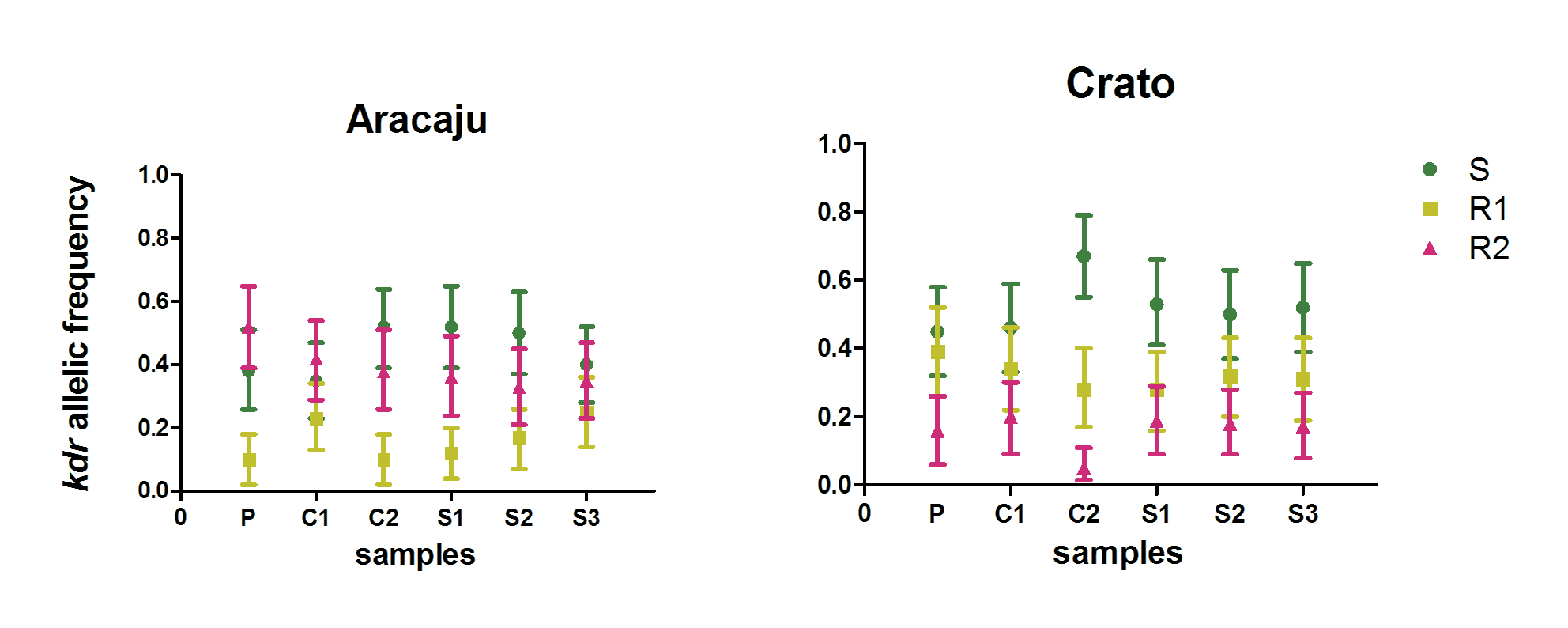

Supplement: S2 Fig — The 95% confidence limit of each one of the alleles S (1016 Val + 1534Phe), R1 (1016 Val + 1534Cys) and R2 (1016Ile + 1534Cys) was plotted for both Aracaju and Crato mosquitoes S: green, R1: yellow, R2: pink. In the x-axis, P, C1-2 and S1-3 refer to, respectively, Parental, Control and Selected samples. (TIF) [file pntd.0006734.s002.tif]
