# Supplementary material for: Laboratory selection of Aedes aegypti field populations with the organophosphate malathion: Negative impacts on resistance to deltamethrin and to the organophosphate temephos
Source: PLoS Negl Trop Dis. 2018 Aug 20;12(8):e0006734. doi: 10.1371/journal.pntd.0006734 (PMC6128625; doi:10.1371/journal.pntd.0006734)
Supplement: S1 Table — Results generated by probit analyses. Results of bioassays with the Rockefeller strain tested simultaneously to the experimental and control groups are also shown. SR: ‘Selection Rate’, ratio between the LC (or EI) values of C and S groups and their corresponding parental population (P). LC: lethal concentration. The 95% confidence interval is shown. (PDF) [file pntd.0006734.s003.pdf]

population	sample	generation	Malathion (larvae) LC_{50} (mg/L)	LC_{95} (mg/L)	confidence intervals LC_{50} (mg/L)	confidence intervals LC_{95} (mg/L)	RR_{50}	RR_{95}	SR_{50}	SR_{95}	*slope*
Rock	.-.	.-.	0.049	0.088	0.04799 < LC < 0.05058	0.08438 < LC < 0.09259	1.0	1.0	.-.	.-.	6.5
Aracaju	P	F2	0.094	0.213	0.09142 < LC < 0.09741	0.21254 < LC < 0.22242	2.6	2.9	1.0	1.0	4.7
	C1	F7	0.120	0.231	0.11608 < LC < 0.12366	0.22036 < LC < 0.24261	2.4	2.6	1.3	1.1	5.8
	C2		0.111	0.221	0.10786 < LC < 0.11502	0.21086 < LC < 0.23243	2.3	2.5	1.2	1.0	5.5
	S1	F7	0.121	0.273	0.11592 < LC < 0.12540	0.25585 < LC < 0.29130	2.5	3.1	1.3	1.3	4.6
	S2		0.172	0.357	0.16647 < LC < 0.17769	0.33785 < LC < 0.37651	3.5	4.0	1.8	1.7	5.2
	S3		0.141	0.339	0.13487 < LC < 0.14754	0.32034 < LC < 0.35847	2.9	3.8	1.5	1.6	4.3
Crato	P	F2	0.086	0.207	0.08350 < LC < 0.08928	0.19702 < LC < 0.21668	2,4	2,8	1.0	1.0	4.3
	C1	F6	0.112	0.234	0.10824 < LC < 0.11576	0.22182 < LC < 0.24612	2.3	2.6	1.3	1.1	5.2
	C2		0.106	0.220	0.10238 < LC < 0.10962	0.20900 < LC < 0.23147	2.2	2.5	1.2	1.1	5.2
	S1	F7	0.262	0.519	0.25458 < LC < 0.27055	0.49096 < LC < 0.54918	5.3	5.9	3.0	2.5	5.6
	S2		0.232	0.454	0.22495 < LC < 0.23923	0.43227 < LC < 0.47663	4.7	5.1	2.7	2.2	5.6
	S3		0.235	0.470	0.22601 < LC < 0.24499	0.44554 < LC < 0.49628	4.8	5.3	2.7	2.3	5.5
