# Supplementary material for: Laboratory selection of Aedes aegypti field populations with the organophosphate malathion: Negative impacts on resistance to deltamethrin and to the organophosphate temephos
Source: PLoS Negl Trop Dis. 2018 Aug 20;12(8):e0006734. doi: 10.1371/journal.pntd.0006734 (PMC6128625; doi:10.1371/journal.pntd.0006734)
Supplement: S2 Table — Legend as in S1 Table. (PDF) [file pntd.0006734.s004.pdf]

| | | | Malathion (adults) | | | | | | | | |
|---|---|---|---|---|---|---|---|---|---|---|---|
| | | | | | confidence intervals | | | | | | |
| population | sample | generation | $LC_{50}$ (g/L) | $LC_{95}$ (g/L) | $LC_{50}$ (g/L) | $LC_{95}$ (g/L) | $RR_{50}$ | $RR_{95}$ | $SR_{50}$ | $SR_{95}$ | *slope* |
| Rock | .-. | .-. | 0.186 | 0.257 | 0.18223 < LC < 0.19058 | 0.24829 < LC < 0.26660 | 1.0 | 1.0 | .-. | .-. | 11.7 |
| Aracaju | P | F1 | 0.294 | 0.522 | 0.27786 < LC < 0.31058 | 0.48529 < LC < 0.56057 | 1.6 | 1.8 | 1.0 | 1.0 | 6.6 |
| | C1 | F7 | 0.320 | 0.630 | 0.29833 < LC < 0.34304 | 0.56280 < LC < 0.70528 | 1.7 | 2.5 | 1.1 | 1.2 | 5.6 |
| | C2 | | 0.303 | 0.661 | 0.28087 < LC < 0.32600 | 0.58213 < LC < 0.74958 | 1.6 | 2.6 | 1.0 | 1.3 | 4.9 |
| | S1 | F7 | 0.342 | 0.612 | 0.32491 < LC < 0.35981 | 0.55591 < LC < 0.67349 | 1.8 | 2.4 | 1.2 | 1.2 | 6.5 |
| | S2 | | 0.380 | 0.695 | 0.36195 < LC < 0.39871 | 0.62586 < LC < 0.77146 | 2.0 | 2.7 | 1.3 | 1.3 | 6.3 |
| | S3 | | 0.357 | 0.610 | 0.34149 < LC < 0.37415 | 0.55820 < LC < 0.66653 | 1.9 | 2.4 | 1.2 | 1.2 | 7.1 |
| Crato | P | F3 | 0.299 | 0.606 | 0.27089 < LC < 0.30218 | 0.56495 < LC < 0.64953 | 1.6 | 2.0 | 1.0 | 1.0 | 5.0 |
| | C1 | F6 | 0.339 | 0.685 | 0.31655 < LC < 0.36227 | 0.61311 < LC < 0.76575 | 1.8 | 2.7 | 1.1 | 1.1 | 5.4 |
| | C2 | | 0.347 | 0.648 | 0.32671 < LC < 0.36804 | 0.57556 < LC < 0.73051 | 1.9 | 2.5 | 1.2 | 1.1 | 6.1 |
| | S1 | F7 | 0.458 | 0.823 | 0.43701 < LC < 0.47985 | 0.74136 < LC < 0.91359 | 2.5 | 3.2 | 1.5 | 1.4 | 6.5 |
| | S2 | | 0.466 | 0.960 | 0.44267 < LC < 0.49157 | 0.86888 < LC < 1.06041 | 2.5 | 3.7 | 1.6 | 1.6 | 5.3 |
| | S3 | | 0.419 | 0.871 | 0.39320 < LC < 0.44749 | 0.78748 < LC < 0.96324 | 2.3 | 3.4 | 1.4 | 1.4 | 5.2 |
