# Supplementary material for: Laboratory selection of Aedes aegypti field populations with the organophosphate malathion: Negative impacts on resistance to deltamethrin and to the organophosphate temephos
Source: PLoS Negl Trop Dis. 2018 Aug 20;12(8):e0006734. doi: 10.1371/journal.pntd.0006734 (PMC6128625; doi:10.1371/journal.pntd.0006734)
Supplement: S3 Table — Legend as in S1 Table. (PDF) [file pntd.0006734.s005.pdf]

population	sample	generation	Temephos								
			LC_{50} (mg/L)	LC_{95} (mg/L)	confidence intervals		RR_{50}	RR_{95}	SR_{50}	SR_{95}	*slope*
					LC_{50} (mg/L)	LC_{95} (mg/L)					
Rock	.-.	.-.	0.005	0.008	0.00524 < LC < 0.00537	0.00820 < LC < 0.00857	1.0	1.0	.-.	.-.	8.3
Aracaju	P	F1	0.040	0.098	0.03858 < LC < 0.04107	0.09273 < LC < 0.10286	11.2	12.9	1.0	1.0	4.2
	C1	F7	0.046	0.096	0.04465 < LC < 0.04760	0.09138 < LC < 0.10113	8.7	11.5	1.2	1.0	5.2
	C2		0.044	0.108	0.04254 < LC < 0.04591	0.10150 < LC < 0.11532	8.3	12.9	1.1	1.1	4.2
	S1	F7	0.031	0.080	0.02928 < LC < 0.03259	0.07510 < LC < 0.08623	5.8	9.6	0.8	0.8	4.0
	S2		0.040	0.087	0.03815 < LC < 0.04164	0.08139 < LC < 0.09202	7.5	10.3	1.0	0.9	4.9
	S3		0.045	0.099	0.04326 < LC < 0.04715	0.09301 < LC < 0.10476	8.5	11.8	1.1	1.0	4.8
Crato	P	F2	0.048	0.332	0.04317 < LC < 0.05412	0.29850 < LC < 0.36883	23.2	64.8	1.0	1.0	2.0
	C1	F6	0.102	0.368	0.09597 < LC < 0.10759	0.33782 < LC < 0.40069	19.1	43.9	2.1	1.1	2.9
	C2		0.071	0.339	0.06589 < LC < 0.07747	0.30658 < LC < 0.37542	13.5	40.4	1.5	1.0	2.4
	S1	F7	0.043	0.223	0.03782 < LC < 0.04903	0.19976 < LC < 0.24943	8.1	26.6	0.9	0.7	2.3
	S2		0.048	0.383	0.04181 < LC < 0.05514	0.33816 < LC < 0.43440	9.1	45.7	1.0	1.2	1.8
	S3		0.066	0.372	0.05880 < LC < 0.07355	0.33274 < LC < 0.41584	12.4	44.3	1.4	1.1	2.2
