# Supplementary material for: Laboratory selection of Aedes aegypti field populations with the organophosphate malathion: Negative impacts on resistance to deltamethrin and to the organophosphate temephos
Source: PLoS Negl Trop Dis. 2018 Aug 20;12(8):e0006734. doi: 10.1371/journal.pntd.0006734 (PMC6128625; doi:10.1371/journal.pntd.0006734)
Supplement: S4 Table — Legend as in S1 Table. EI: Emergence inhibition. (PDF) [file pntd.0006734.s006.pdf]

population	sample	generation	Diflubenzuron								
			EI_{50} (μg/L)	EI_{95} (μg/L)	confidence intervals EI_{50} (μg/L)	confidence intervals EI_{95} (μg/L)	RR_{50}	RR_{95}	SR_{50}	SR_{95}	*slope*
Rock	.-.	.-.	1.717	2.978	1.64664 < LC < 1.78964	2.75355 < LC < 3.22043	1.0	1.0	.-.	.-.	6.9
Aracaju	P	F1	1.574	4.031	1.49646 < LC < 1.65528	3.64624 < LC < 4.45778	1.6	1.7	1.0	1.0	4.0
	C1	F7	2.364	3.837	2.26826 < LC < 2.46480	3.57900 < LC < 4.11358	1.4	1.3	1.5	1.0	7.8
	C2		2.131	4.663	2.01306 < LC < 2.25560	4.23165 < LC < 5.13764	1.2	1.6	1.4	1.2	4.8
	S1	F7	2.322	5.475	2.19893 < LC < 2.45230	4.94692 < LC < 6.05879	1.4	1.8	1.5	1.4	4.4
	S2		2.419	4.721	2.32590 < LC < 2.51625	4.38859 < LC < 5.07868	1.4	1.6	1.5	1.2	5.7
	S3		2.675	5.805	2.54809 < LC < 2.80739	5.25405 < LC < 6.41321	1.6	1.9	1.7	1.4	4.9
Crato	P	F2	2.002	3.982	1.91585 < LC < 2.09109	3.70419 < LC < 4.27966	1.6	1.9	1.0	1.0	5.5
	C1	F6	2.164	3.957	2.06379 < LC < 2.26832	3.64920 < LC < 4.29096	1.3	1.3	1.1	1.0	6.3
	C2		2.006	3.670	1.91027 < LC < 2.10735	3.39976 < LC < 3.96193	1.2	1.2	1.0	0.9	6.3
	S1	F7	2.511	5.497	2.39073 < LC < 2.63696	5.00953 < LC < 6.03289	1.5	1.9	1.3	1.4	4.8
	S2		3.083	4.964	2.97765 < LC < 3.19215	4.67612 < LC < 5.26874	1.8	1.7	1.5	1.2	7.9
	S3		2.724	4.954	2.60888 < LC < 2.84385	4.60621 < LC < 5.32709	1.6	1.7	1.4	1.2	6.3
