# Supplementary material for: Laboratory selection of Aedes aegypti field populations with the organophosphate malathion: Negative impacts on resistance to deltamethrin and to the organophosphate temephos
Source: PLoS Negl Trop Dis. 2018 Aug 20;12(8):e0006734. doi: 10.1371/journal.pntd.0006734 (PMC6128625; doi:10.1371/journal.pntd.0006734)
Supplement: S5 Table — Legend as in S1 Table. (PDF) [file pntd.0006734.s007.pdf]

			Deltamethrin								
population	sample	generation	LC_{50} (mg/L)	LC_{95} (mg/L)	confidence intervals LC_{50} (mg/L)	confidence intervals LC_{95} (mg/L)	RR_{50}	RR_{95}	SR_{50}	SR_{95}	*slope*
Rock	.-.	.-.	0.755	2.717	0.70248 < LC < 0.81079	2.36109 < LC < 3.12665	1.0	1.0	.-.	.-.	3.0
Aracaju	P	F1	13.145	37.784	12.4253 < LC < 13.9069	33.5690 < LC < 42.5283	14.3	17.8	1.0	1.0	3.6
	C1	F7	30.728	163.599	27.44590 < LC < 34.40363	126.26339 < LC < 211.97545	40.7	60.2	2.3	4.3	2.3
	C2		31.558	245.824	27.47851 < LC < 36.24265	172.37382 < LC < 350.57140	41.8	90.5	2.4	6.5	1.9
	S1	F7	8.739	62.534	7.40299 < LC < 10.31635	48.27281 < LC < 81.00770	11.6	23.0	0.7	1.7	1.9
	S2		7.329	46.761	6.11714 < LC < 8.78209	37.61250 < LC < 58.13428	9.7	17.2	0.6	1.2	2.0
	S3		8.263	44.664	7.10054 < LC < 9.61691	36.47431 < LC < 54.69278	11.0	16.4	0.6	1.2	2.2
Crato	P	F3	27.912	140.201	25.5326 < LC < 30.5141	120.1620 < LC < 163.5812	37.0	51.6	1.0	1.0	2.3
	C1	F6	29.888	247.729	25.31606 < LC < 35.28678	175.14364 < LC < 350.39651	39.6	91.2	1.1	1.8	1.8
	C2		30.796	204.656	26.12587 < LC < 36.30162	151.93926 < LC < 275.66375	40.8	75.3	1.1	1.5	2.0
	S1	F7	21.393	95.144	19.11348 < LC < 23.94396	76.67948 < LC < 118.05579	28.4	35.0	0.8	0.7	2.5
	S2		19.923	111.709	17.69118 < LC < 22.43599	88.21355 < LC < 141.46266	26.4	41.1	0.7	0.8	2.2
	S3		24.276	102.520	22.03141 < LC < 26.74946	84.02099 < LC < 125.09306	32.2	37.7	0.9	0.7	2.6
