# Supplementary material for: Laboratory selection of Aedes aegypti field populations with the organophosphate malathion: Negative impacts on resistance to deltamethrin and to the organophosphate temephos
Source: PLoS Negl Trop Dis. 2018 Aug 20;12(8):e0006734. doi: 10.1371/journal.pntd.0006734 (PMC6128625; doi:10.1371/journal.pntd.0006734)
Supplement: S6 Table — (PDF) [file pntd.0006734.s008.pdf]

population	sample	generation	1016 allelic frequency	1016 genotypic frequency	1534 allelic frequency	1534 genotypic frequency
Aracaju	(*)	F0	0.52	0.30	0.62	0.43
	C1	F7	0.42	0.10	0.65	0.40
	C2		0.38	0.10	0.48	0.20
	S1	F7	0.36	0.07	0.47	0.13
	S2		0.33	0.07	0.50	0.27
	S3		0.35	0.07	0.60	0.30
Crato	(*)	F2	0.17	0.03	0.55	0.31
	C1	F6	0.20	0.00	0.53	0.23
	C2		0.05	0.00	0.33	0.17
	S1	F7	0.19	0.00	0.47	0.13
	S2		0.18	0.03	0.50	0.13
	S3		0.17	0.07	0.50	0.37
