# Supplementary material for: Laboratory selection of Aedes aegypti field populations with the organophosphate malathion: Negative impacts on resistance to deltamethrin and to the organophosphate temephos
Source: PLoS Negl Trop Dis. 2018 Aug 20;12(8):e0006734. doi: 10.1371/journal.pntd.0006734 (PMC6128625; doi:10.1371/journal.pntd.0006734)
Supplement: S7 Table — Samples' median values were compared with the corresponding medians of parental strains [29] using the Kruskal-Wallis test. (*): significantly different values (p<0.01). n1: number of individuals considered in the analysis. med2: median of enzymatic activities. (PDF) [file pntd.0006734.s009.pdf]

	samples		AChI		AChE		MFO		α-Est		β-Est		ρnpa-Est		GST	
			n[1]	med[2]	n	med	n	med	n	med	n	med	n	med	n	med
Aracaju (larvae)	**Par**	F1	137	11.82	137	0.07	137	43.23	137	19.22	137	28.50	132	4.40	137	0.71
	**C1**	F7	85	10.53	85	0.05	85	48.89	85	20.05	85	25.49	76	3.22	85	0.70
	**C2**		84	9.86	86	0.04	86	39.58	86	21.28	86	28.40	83	3.02	85	0.73
	**C1+C2**		169	10.26	171	0.04	171	43.93	171	20.53	171	26.57	159	3.12	170	0.71
	**S1**	F7	80	11.62	80	0.06	79	31.28	80	14.08	80	24.81	78	3.74	80	0.88
	**S2**		79	11.21	79	0.05	79	43.52	79	19.03	79	26.36	76	3.48	79	0.64
	**S3**		77	10.02	79	0.05	80	43.86	79	16.48	80	30.87	64	2.97	80	0.67
	**S1+S2+S3**		236	10.89	238	0.05	238	40.17	238	16.34	239	26.73	218	3.42	239	0.70
Aracaju (adults)	**Par**	F1	117	22.41	117	0.15	116	56.68	118	8.73	118	7.67	111	5.73	111	0.94
	**C1**	F7	67	15.91	69	0.14	69	80.62*	68	7.11	69	6.17	61	4.79	69	1.50*
	**C2**		70	17.54	70	0.13	70	82.33*	70	7.90	70	7.37	68	6.90	69	1.41*
	**C1+C2**		137	16.98	139	0.13	139	82.56*	138	7.57	139	6.84	129	5.91	138	1.45*
	**S1**	F7	65	14.25	65	0.15	64	68.12*	65	6.99	59	6.18	62	5.67	63	1.23*
	**S2**		68	16.58	70	0.12	70	67.12*	68	7.35	70	6.95	67	5.77	54	1.15*
	**S3**		70	15.92	70	0.15	70	77.35*	69	7.88	70	7.51	69	5.11	69	1.26*
	**S1+S2+S3**		203	15.95	205	0.14	204	72.71*	202	7.47	199	7.06	198	5.15	186	1.19*
Crato (larvae)	**Par**	F2	92	12.39	92	0.06	91	31.43	92	14.36	92	28.14	91	3.73	92	0.75
	**C1**	F6	79	11.34	80	0.05	79	40.47*	80	21.37*	80	28.79	75	3.05	80	0.60
	**C2**		80	11.16	80	0.04	80	36.40	80	20.47*	79	26.22	76	2.75	80	0.52
	**C1+C2**		159	11.23	160	0.04	159	37.55	160	20.83*	159	27.46	151	2.89	160	0.57
	**S1**	F7	78	9.24	79	0.06	78	40.22*	79	20.82*	79	29.61	69	3.00	79	0.84
	**S2**		77	9.22	77	0.05	78	36.12	79	22.28*	79	30.71	69	3.03	79	0.69
	**S3**		80	8.79	80	0.05	79	38.83*	80	20.02*	80	33.42*	72	3.07	80	0.88
	**S1+S2+S3**		235	9.17	236	0.05	235	38.83*	238	21.17*	238	30.95	210	3.04	238	0.81
Crato (adults)	**Par**	F2	171	17.06	174	0.14	120	32.05	174	6.63	173	6.14	161	4.03	163	1.16
	**C1**	F6	69	18.71	69	0.12	69	79.38*	69	9.10*	69	9.01*	68	8.01*	69	1.41*
	**C2**		68	15.53	70	0.13	70	81.44*	70	8.66*	69	8.44*	68	7.09*	69	1.22
	**C1+C2**		137	17.12	139	0.12	139	83.34*	139	8.73*	138	8.99*	136	7.76*	138	1.30*
	**S1**	F7	80	17.35	80	0.13	80	63.37*	80	8.96*	79	8.20*	77	7.86*	79	1.36*
	**S2**		70	17.87	70	0.11	70	75.28*	70	10.24*	70	10.31*	68	9.11*	70	1.31*
	**S3**		69	16.84	70	0.14	69	77.46*	70	8.72*	69	8.31*	69	6.65*	69	1.36*
	**S1+S2+S3**		219	17.32	220	0.13	219	77.99*	220	9.54*	218	9.21*	214	7.60*	218	1.35*
